# Supplementary material for: Prognostic Ability of Tumor Budding Outperforms Poorly Differentiated Clusters in Gastric Cancer
Source: Cancers (Basel). 2022 Sep 28;14(19):4731. doi: 10.3390/cancers14194731 (PMC9563769; doi:10.3390/cancers14194731)
Supplement: Supplementary file 1 [file cancers-14-04731-s001.zip › cancers-1917444-supplementary.pdf]

Supplementary Materials

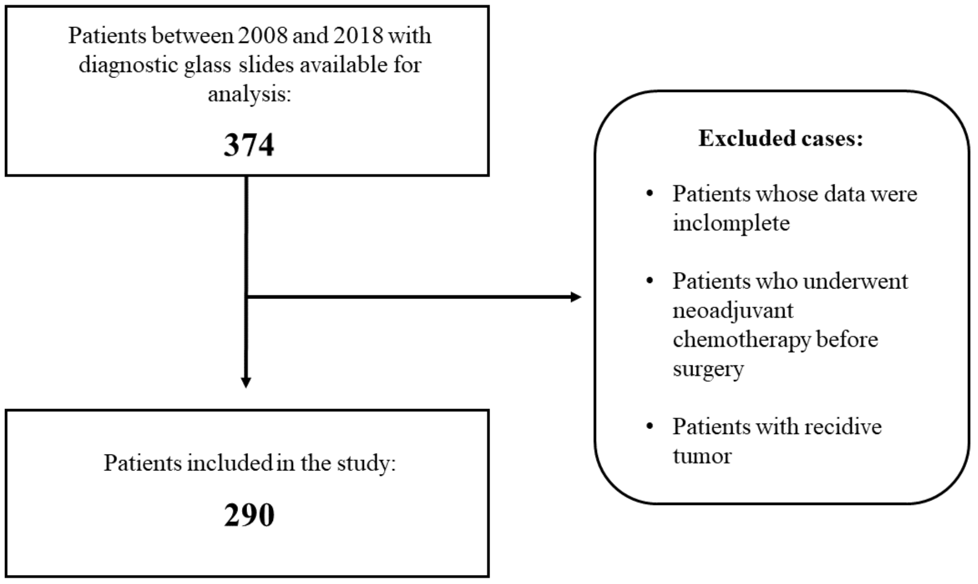

Supplementary Figure S1 – Case selection of the study.

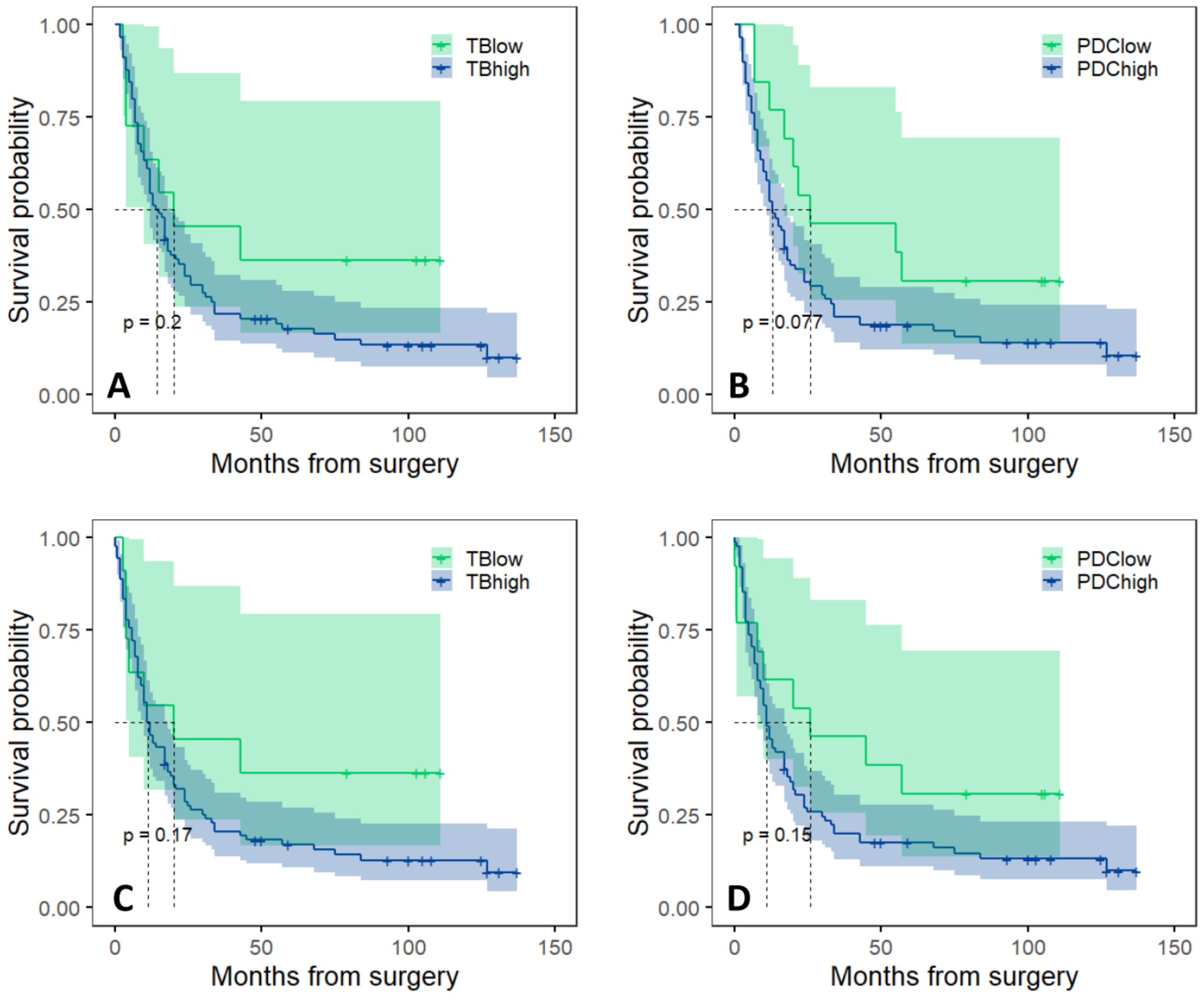

Supplementary Figure S2 - Kaplan-Meier curves of OS and DFS survival analyses in diffuse type adenocarcinomas - A) OS by TB low and TB high groups, B) OS by PDC low and PDC high groups, C) DFS by TB low and TB high groups, D) DFS by PDC low and PDC high groups in diffuse type adenocarcinomas. (TB: tumor budding, PDC: poorly differentiated cluster, OS: overall survival, DFS: disease free survival)

Supplementary Table S1 - Univariable and multivariable survival analyses in diffuse type adenocarcinomas. (pT: Primary tumor extent, pN: Regional lymph node metastasis, pM: Distant metastasis, TB: tumor budding, PDC: poorly differentiated cluster, n.s.: statistically non-significant; Statistically significant *p* values are displayed in bold)

**Parameter**		**Diffuse type - Overall Survival**				**Diffuse type - Disease Free Survival**			
		**Univariable**		**Multivariable**		**Univariable**		**Multivariable**	
		HR (95% CI)	*p*	HR (95% CI)	*p*	HR (95% CI)	*p*	HR (95% CI)	*p*
TB	low/high	1.7 (0.76-3.6)	0.2	n.s.	n.s.	1.7 (0.86-3.4)	0.12	n.s.	n.s.
PDC	low/high	1.9 (0.93-3.7)	0.082	n.s.	n.s.	1.4 (0.74-2.6)	0.3	n.s.	n.s.
Sex	M/F	0.82 (0.53-1.3)	0.36	n.s.	n.s.	0.72 (0.49-1.1)	0.1	n.s.	n.s.
Age	years	1 (1-1.1)	0.012	n.s.	n.s.	1 (1-1)	0.058	n.s.	n.s.
pT	I-II/ III-IV	6.2 (1.9-20)	0.002	5.846 2.362-14.47	**0.0001**	6.1 (2.5-15)	<0.0001	6.52 (2.63-16.16)	**<0.0001**
pN	pN0/pN+	4.4 (1.9-10)	0.0006	n.s.	n.s.	4.6 (2.3-9.1)	<0.0001	n.s.	n.s.
pM	pM0/pM1	1.8 (0.81-3.9)	0.15	n.s.	n.s.	2.6 (1.4-5.1)	0.0041	3.16 (1.63-6.16)	**0.0007**
Grade	G1/G2/G3	1.2 (0.43-3.2)	0.76	n.s.	n.s.	1.7 (0.75-3.9)	0.2	n.s.	n.s.
Residual tumor	absent/ present	1.6 (0.97-2.5)	0.066	n.s.	n.s.	1.8 (1.2-2.8)	0.0069	n.s.	n.s.
Lymphovascular invasion	absent/ present	2.0 (1.2-3.4)	0.011	n.s.	n.s.	2 (1.2-3.1)	0.0047	n.s.	n.s.
Perineural invasion	absent/ present	0.98 (0.62-1.6)	0.94	n.s.	n.s.	1.3 (0.86-1.9)	0.23	n.s.	n.s.
